# Differentiation of Breast Lesions and Distinguishing Their Histological Subtypes Using Diffusion-Weighted Imaging and ADC Values

**DOI:** 10.3389/fonc.2020.00332

**Published:** 2020-03-13

**Authors:** Jelena Maric, Jasmina Boban, Tatjana Ivkovic-Kapicl, Dragana Djilas, Viktorija Vucaj-Cirilovic, Dragana Bogdanovic-Stojanovic

**Affiliations:** ^1^General Hospital “Sveti Vračevi”, Bijeljina, Bosnia and Herzegovina; ^2^Faculty of Medicine Novi Sad, University of Novi Sad, Novi Sad, Serbia; ^3^Center for Diagnostic Imaging, Oncology Institute of Vojvodina, Sremska Kamenica, Serbia; ^4^Department for Pathology, Oncology Institute of Vojvodina, Sremska Kamenica, Serbia

**Keywords:** breast cancer, magnetic resonance imaging, Diffusion-weighted (DW) imaging, ADC values, differentiation

## Abstract

Diffusion-weighted imaging (DWI) has not been well explored in differentiation of malignant from benign breast lesions. The aims of this study were to examine the role of apparent diffusion coefficient (ADC) values in differentiation of malignant from benign tumors and distinguishing histological subtypes of malignant lesions, and to determine correlations between ADC values and breast tumors structure. This cohort-study included 174 female patients who underwent contrast-enhanced breast MR examination on a 3T scanner and were divided into two groups: patient group (114 patients with proven tumors) and control group (60 healthy patients). One-hundred-thirty-nine lesions (67 malignant and 72 benign) were detected and pathohistologically analyzed. Differences between variables were tested using chi-square test; correlations were determined using Pearson's correlation test. For determination of cut off values for diagnostic potential, Receiver Operating Characteristic curves were constructed. Statistical significance was set at *p* < 0.05. Mean ADC values were significantly lower in malignant compared to benign lesions (0.68 × 10^−3^mm^2^/s vs. 1.12 × 10^−3^mm^2^/s, *p* < 0.001). The cut off value of ADC for benign lesions was 0.792 × 10^−3^mm^2^/s (sensitivity 98.6%, specificity 65.7%), and for malignant 0.993 × 10^−3^mm^2^/s (98.5, 80.6%). There were no significant correlations between malignant lesion subtypes and ADC values. DWI is a clinically useful tool for differentiation of malignant from benign lesions based on mean ADC values. The cut off value for benign lesions was higher than reported recently, due to high amount of fibrosis in included benign lesions. Finally, ADC values might have implications in determination of the biological nature of the malignant lesions.

## Introduction

Magnetic resonance imaging (MRI) of the breast has an important role in detection, evaluation and follow-up of breast lesions. This diagnostic modality is based both on the analysis of morphologic parameters (available from conventional, native MRI) and the kinetic features of the lesion (available from dynamic contrast-enhanced (DCE) study) (1). Abbreviated MRI protocols represent a novel approach to diagnostics and screening of the breast lesions, tailored to achieve similar diagnostic accuracy as conventional protocols but in a considerably shorter amount of time spent for the acquisition (2, 3). The role of native and DCE study in these abbreviated protocols has been well-described. However, the role of other advanced MRI techniques—diffusion-weighted imaging in the first place—has not been well-explored to date.

Diffusion-weighted imaging (DWI) represents an MRI technique that depicts Brownian motion of water molecules, indirectly showing the degree of tissue cellularity and integrity of cell membranes (4). Breast cancers typically present with restricted diffusion of water molecules, observed as increase in DWI signal, and lower apparent diffusion coefficient (ADC) values compared to normal surrounding tissue and benign lesions of the breast (5). However, there are some exceptions to this observation. Namely, some benign breast lesions show low apparent diffusion coefficient (ADC) values, while ductal carcinoma *in situ* (DCIS) can show higher ADC values than invasive carcinoma.

The aim of this study was to explore the possibilities of ADC values in differentiation of malignant from benign breast tumors. The secondary aim was to explore the possibilities of DWI and ADC values in distinguishing histological subtypes of breast malignant lesions. The final aim was to determine the correlations between ADC values and structure of the breast tissue based on ACR classification.

## Materials and Methods

### Subject Selection

This retrospective observational cohort-study was conducted on a total of 174, randomly selected female patients who were referred to MR examination of the breast in the period January 2013–2017. The study was approved by the institutional ethical committee. When indicated, percutaneous biopsies or surgical procedures were performed after imaging examination in a very close period of time (up to 3 weeks).

The inclusion criteria for this study were: age over 18, female gender. The exclusion criteria were: the absence of prior mammographic examination, contraindications for MRI examination for both groups, while the additional exclusion criterion for the patient group was the absence of subsequent histological finding.

Patients were divided into two groups: the first group consisted of 114 patients with pathohistologically verified tumors in the breast, and the second group was the control group, consisted of 60 healthy patients with no intraparenchymal pathologic changes (MRI and digital mammography verified). Control group was formed of patients who underwent MR mammography for the purpose of screening. In the first group, an overall of 139 lesions was detected and analyzed (72 benign lesions in 64 patients and 69 malignant lesions in 50 patients). All patients signed a fully informed written consent to take part in this study.

### MRI Examination

**Figure 1:**
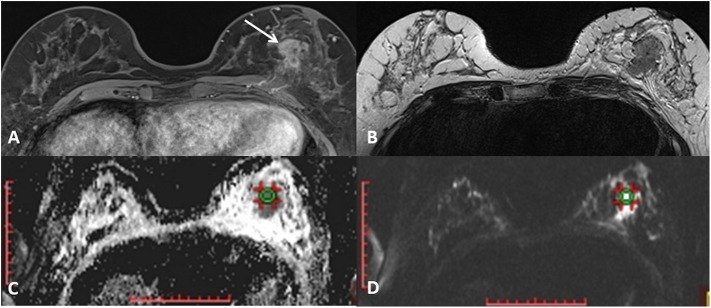
A mass lesion is identified on the first post-contrast T1-weighted image **(A)** and the absence of cystic parts is confirmed on T2-weighted axial image **(B)**. ROI of 1cm^3^ is defined on correspondent DWI image **(C)** and the ADC values are noted **(D)**.

MR mammography in all patient was performed on the same 3T MR unit (Siemens Trio Tim, Erlangen, Germany), using a dedicated 36-channel coil, in the prone position. Conventional MR protocol included non-fat-suppressed T2-weigthed turbo spin echo transversal, non-fat-suppressed and fat suppressed T1-weighetd transversal sequences and STIR sagittal sequence, followed by dynamic contrast study (fat-suppressed 3D T1-weighted Fast Low Angle SHot (FLASH) transversal tomograms). Gadolinium contrast agent was injected in the dose of 0.1 mmol/kg, at the rate 2.5 ml/s, followed by 25 ml saline injection. Parameters for dynamic contrast study were: time of repetition/ time of echo (TR/TE) 4.2 ms/1.6 ms, flip angle (FA) 15°, field of view (FOV) 340 × 340 mm, matrix size 512 × 410, slice thickness 2 mm, time of acquisition 86 s. Diffusion-weighted imaging was performed prior to contrast study, using echo-planar imaging (EPI) sequence in the axial plane, with 4*b* values: of 250, 500, 750, and 1500 s/mm^2^. Parameters for this sequence were: TR/TE 5489,3 ms/89 ms, FOV 340 × 170 mm, matrix size 192 × 96, slice thickness 4 mm, scanning time 67 s. Echo time was chosen according to the “optimal TE” option, that automatically estimates the shortest echo time for the diffusion-weighted sequence. Apparent diffusion coefficient (ADC) maps were constructed during the post-processing, using commercially available software provided by the manufacturer (Syngo, Siemens Healthcare). The region of interest (ROI) selection was performed manually, in the solid parts of tumors identified on the anatomical images (the first T1 post-contrast image, and subtraction images), avoiding cystic components, and then correlated with the position on the DWI tomograms (Figure 1). The typical ROI size was 1 cm^3^, and it was delineated on a single slice. Morphologic and kinetic features of the lesions were assessed on dynamic MRI study and classified according to the BI RADS classification system (6). Mammographic structure of the breast was assessed according to the guidelines provided by ACR, and classified into 4 groups (A to D) according to the subjectively assessed distribution of fibroglandular and fat tissue in the breast.

### Pathohistological Evaluation

Final diagnosis was established based on histological examination, and tissue samples were obtained using percutaneous biopsies (core biopsy) or surgical excision. Core biopsy was performed using Bard Magnum biopsy instrument and needles of 14 G. Three to five tissue samples were taken and put into formalin. Pathohistological report provided the lesion type (benign/malignant) and histological finding according to the WHO Classification of the breast tumors and Classification of the benign lesions of the breast (7). Hormone receptor status was determined using immunohistochemistry, and Her2 retest in undefined 2+ status using “*in situ*” hybridization. For determination of the hormone receptor status, the well-known Allred score was used. It is based on two parameters: the percentage of cells stained in the sample (values 1–5) and on the staining intensity (1, 2, or 3). The hormone receptor status is negative if Allred score is 0–2, slightly positive if 3–5 and positive if 6–8 (8).

### Statistical Analysis

Statistical analysis was performed using SPSS ver. 19.0 (IBM, Chicago, IL, USA). Methods of descriptive statistics were used (mean, standard deviation, minimum, maximum, or median and interquartile range, depending on the variable type), and the distribution was tested using Kolmogornov–Smirnov test. Differences between variables (age, ACR category, BI RADS category, mean ADC value, hormone receptors state) were tested using chi-square test since it is not dependent on the distribution of data. Correlations were determined using Pearson's correlation test. Univariate analysis of variance (ANOVA) was used for determination of the differences between mean values of the variables with normal distribution in both groups (e.g., age difference). For determination of cut off values for diagnostic and prognostic potential of variables, Receiver Operating Characteristic (ROC) curves were constructed, with defining sensitivity, specificity, positive and negative predictive values using standard formulas.

Statistical significance was set at value *p* < 0.05.

## Results

Mean age in the study group was 43.53 ± 10.81 (range 22–82), while in the control group it was 55.73 ± 9.58 years (range 31–75). There was significant difference between two groups regarding age, with controls significantly older (*p* < 0.001, *F* = 8.695).

Mammographic and MR mammographic breast tissue density results were determined for all the lesions: the most common type in both groups was ACR B (53.33% of the control group and 47.37% of the study group). In the control group, the most common MR mammography type of the parenchymal background enhancement was ACR A (55%), while in study group it was ACR B (40.35%).

**Figure 2:**
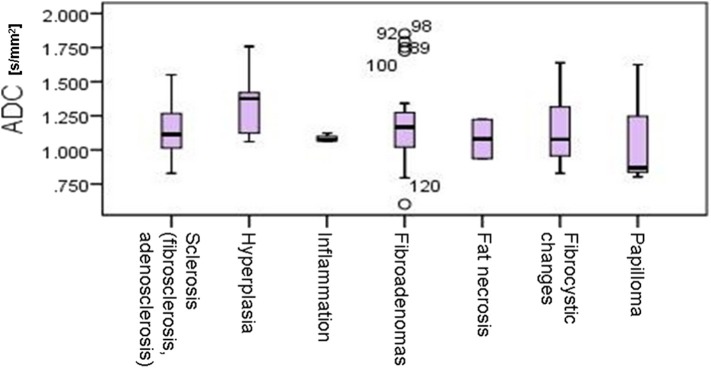
Differences in the ADC values between different benign lesion types.

**Figure 3:**
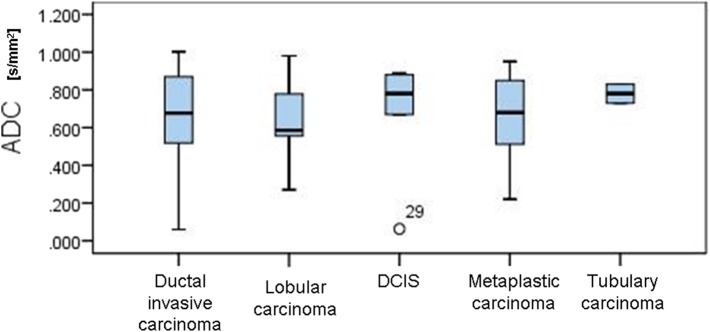
Differences in the ADC values between different malignant lesions.

In the control group, all MR mammograms were classified as BI RADS 1 category. In the study group, 11 lesions were classified as BI RADS 2 (7.91%), 6 lesions as BI RADS 3 (4.32%), 36 lesions as BI RADS 4 (25.9), 39 lesions as BI RADS 5 (28.06%), and 6 lesions were classified as BI RADS 6 (13.67%). Pathohistological evaluation revealed 67 malignant (48.2%) and 72 (51.8%) lesions. Mean ADC values and distribution of pathohistologic diagnoses in all benign and malignant lesions are summarized in Table 1. Since in some categories of the benign lesions there were only 2 or 3 cases, these lesions were excluded from further statistical analysis. Differences in the ADC values between different benign lesion types are shown on Figure 2, and between different malignant lesions on Figure 3. ADC values were significantly lower in malignant lesions compared to benign (mean ADC in malignant lesions was 0.68 × 10^−3^mm^2^/s, mean ADC in benign 1.12 × 10^−3^mm^2^/s, *p* < 0.001). There were no significant differences in mean ADC values of the different ACR types observed both on mammograms and MR mammograms in the group with benign lesions, and in the group with malignant lesions (Table 2).

**Table 1:** Mean ADC values and distribution of pathohistologic diagnoses in all benign and malignant lesions.

**Lesion type**	**Pathohistological finding**	***N* (frequency)**	**ADC (×10^**−3**^ mm^**2**^/s) (3^**rd**^-1^**st**^ quartile)**	**Max**	**Min**
Benign	Sclerosis (adenosclerosis, fibrosclerosis)	17 (23.6%)	1.114 (1.29–0.75)	1.55	0.83
	Hyperplasia	5 (6.9%)	1.38 (1.59–0.92)	1.76	1.06
	Inflammation	3 (4.2%)	1.07 (1.27–1.02)	1.12	1.06
	Fibroadenoma	32 (44.4%)	1.17 (1.27–1.02)	1.85	0.60
	Fat necrosis	2 (2.8%)	1.08 (1.13–0.94)	1.23	0.94
	Fibrocystic changes	10 (13.9%)	1.08 (1.32–0.94)	1.64	0.83
	Papilloma	3 (4.2%)	0.87 (1.45–0.82)	1.625	0.80
Malignant	Ductal invasive carcinoma	40 (59.9%)	0.68 (0.87–0.52)	1.00	0.06
	Lobular carcinoma	12 (17.9%)	0.72 (0.82–0.55)	0.98	0.27
	DCIS	6 (9%)	0.78 (0.88–0.52)	0.89	0.63
	Metaplastic carcinoma	7 (10.5%)	0.68 (0.85–0.50)	0.95	0.22
	Tubulary carcinoma	2 (3%)	0.78 (0.81–0.75)	0.83	0.73

**Table 2:** Mean ADC values for different ACR types observed on mammograms and MR mammograms in benign and malignant lesions.

**Digital mammography**			**MR mammography**		
**ACR category**	**ADC (×10^**−3**^ mm^**2**^/s) median (1^**st**^-3^**rd**^ quartile)**	***P***	**ACR category**	**ADC (×10^**−3**^ mm^**2**^/s) median (1^**st**^-3^**rd**^ quartile)**	***p***
1	1.17 (1.02–1.29)	0.362	1	1.09 (0.99–1.21)	0.293
2	1.12 (0.96–1.25)		2	1.14 (1.01–1.32)	
3	1.15 (1.01–1.39)		3	1.12 (1.01–1.37)	
4	0.52 (0.43–0.79)	0.091	4	0.67 (0.50–0.83)	0.308
5	0.69 (0.56–0.87)		5	0.69 (0.55–0.87)	
6	0.70 (0.55–0.90)		6	0.83 (0.60–0.94)	

**Figure 4:**
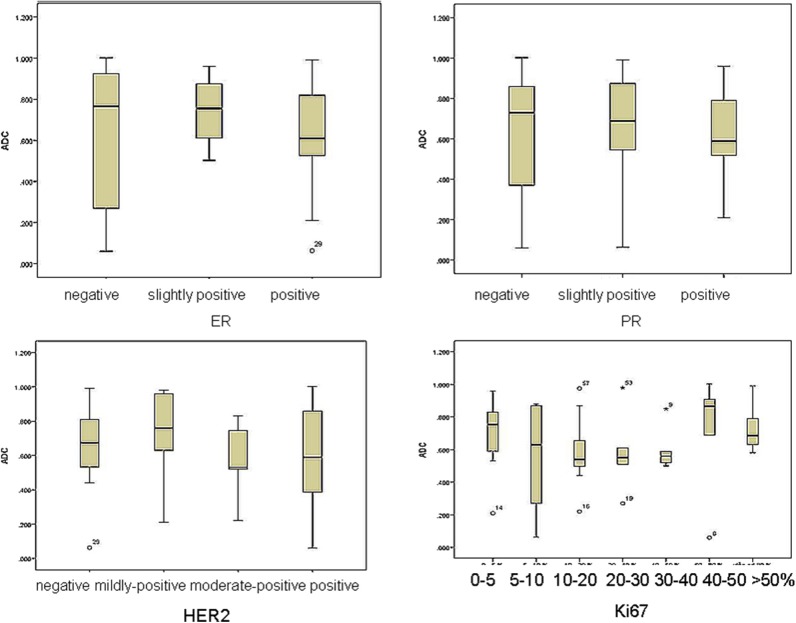
The distribution of mean ADC values in correlation with estrogen, progesterone and Her2 receptor status, as well as with Ki-67 index.

The distribution of mean ADC values in correlation with estrogen, progesterone and Her2 receptor status, as well as with Ki-67 index is shown on Figures 4A–D. Correlations between hormone receptor status and ADC values are summarized in Table 3. The mean ADC values in different molecular types of breast cancer are shown in Table 4.

**Table 3:** Correlations between hormone receptor status and ADC values.

**Parameter**	**Variable**	**ER**	**PR**	**Her2**	**Ki-67**	**Mean ADC value**
ER	Pearson correlation	1	0.537^**^	−0.307^*^	0.047	−0.131
	Sig. (2-tailed)		0	0.017	0.725	0.302
	N	64	64	60	58	64
PR	Pearson correlation	0.537^**^	1	−0.295^*^	0.025	−0.154
	Sig. (2-tailed)	0		0.022	0.854	0.226
	N	64	64	60	58	64
Her2	Pearson correlation	−0.307^*^	−0.295^*^	1	−0.033	−0.117
	Sig. (2-tailed)	0.017	0.022		0.809	0.372
	N	60	60	60	56	60
Ki-67	Pearson correlation	0.047	0.025	−0.033	1	0.156
	Sig. (2-tailed)	0.725	0.854	0.809		0.243
	N	58	58	56	58	58
Mean ADC value	Pearson Correlation	−0.131	−0.154	−0.117	0.156	1
	Sig. (2-tailed)	0.302	0.226	0.372	0.243	
	N	64	64	60	58	139

** *Significance is at the level of 0.01 (2-tailed)*.

* *Significance is at the level 0.05 (2-tailed)*.

*ER, estrogen receptor; PR, progesteron receptor; ADC, apparent diffusion coefficient*.

**Table 4:** The mean ADC values in different molecular types of breast cancer.

**Molecular type of breast cancer**	**ADC (×10^**−3**^ mm^**2**^/s) median (1^**st**^– 3^**rd**^ quartile)**	***p***
Luminal A	0.55 (0.51–0.81)	0.065
Luminal B	0.67 (0.52–0.82)	
Basaloid	0.88 (0.81–0.96)	
Her 2 positive	0.56(0.22–0.90)	

**Figure 5:**
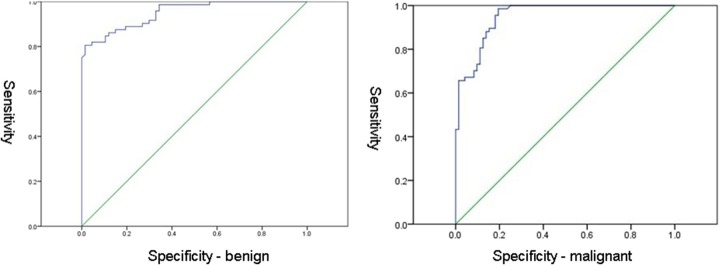
ROC curves for benign **(Left)** and malignant **(Right)** lesions.

ROC curves for benign and malignant lesions are shown in Figure 5. The cut off value of ADC for benign lesions was 0.792 × 10^−3^mm^2^/s (sensitivity 98.6%, specificity 65.7%). The cut off value of ADC for malignant lesions was 0.993 × 10^−3^mm^2^/s (sensitivity 98.5%, specificity 80.6%).

## Discussion

Recent studies confirmed that integration of diffusion-weighted imaging and determination of ADC values added to the differentiation between benign and malignant breast lesions (9, 10). The signal of the lesion observed on DWI is highly influenced by the cellularity of the tumor and permeability of the basal membrane, thus giving some insight into biological features of the lesion (11–13). Moreover, some authors showed that there was a potential significant correlation between ADC values and several prognostic factors, such as tumor size, nuclear grade, biological markers and the presence of pathological lymph nodes (12–14).

According to the results of our study, mean ADC values observed in benign lesions were significantly higher than those observed in malignant lesions, concordantly to the current body of evidence (15–17). This fact makes DWI and determination of ADC values a clinically useful additional tool for differentiation of the lesion nature in every day clinical practice. The lowest ADC values were observed in invasive ductal carcinoma (0.68 × 10^−3^ mm^2^/s), concordantly with recent studies, where the lowest ADC values were observed in invasive ductal and cribriform carcinoma (0.78 × 10^−3^ mm^2^/s), while the highest were observed in micropapillary carcinoma, probably as a result of its typical pathological structure based on the proliferation of tumor cell batches with empty spaces of stroma (15, 18). The highest ADC values in malignant lesions in our study were observed in tubulary carcinoma (0.78 × 10^−3^ mm^2^/s).

The cut off values of mean ADC were determined for both benign and malignant lesions in our study. The cut off ADC value for malignant lesions was 0.792 (sensitivity 98.6%, specificity 65.7%), while the cut off value of mean ADC for benign lesions was 0.993 (sensitivity 98.5%, specificity 80.6%). The cut off value for the benign lesions in our study was lower than those reported in the literature, 1.1-1.6 × 10^−3^ mm^2^/s (15). However, the acquisition of DWI series was different in this study, which was performed on 1.5T scanner, with one *b*-value (750 mm^2^/s). It would be useful to define a “determination line” od a “determination ADC value” that would be able to separate malignant form benign lesions in everyday routine workup. However, in our study, a variety of benign lesions (including adeno- and fibrosclerosis, and inflammation) was included in the analysis, out of which some presented with high amount of fibrous tissue, that could interfere with the calculations of ADC values making them lower than usually observed (19, 20). In our opinion, in routine clinical practice, it is important to be aware that certain histological features of benign lesions (such as extensive fibrosis) may alter the findings on DWI and lead to misdiagnosis if only this parameter was taken into account.

Measurements of ADC are reportedly influenced by the degree of cell proliferation and cell density, thus related to tumor aggressiveness (21). The Ki-67 index reflects the degree of cell proliferation and should negatively correlate with ADC values (the higher the Ki-67, the lower ADC value in the tumor). Previous studies reported that ER-positivity was related to higher tumor cellularity and, thus, the negative correlation with ADC values was expected (22, 23). The same correlation was proposed for PR-positivity in some of the previous studies (24, 25).

In our study, no significant correlations were observed between ADC values and estrogen receptor positive status (χ^2^ = 2.731, *p* = 0.255). However, *p*-value reached significance in the group of highly positive ER (*p* = 0.048), meaning that with the expansion of the study sample and inclusion of the higher number of ER-highly positive lesions, the observed correlations might also change.

We observed no significant correlations between progesterone receptor positive status and ADC values (χ^2^ = 2.948, *p* = 0.229). However, the correlations reached statistical significance in the group of PR-negative tumors (*p* = 0.022). In the study by Park and coauthors, no significant correlations were observed between estrogen receptor status and ADC values (26). Kim et al. also reported no significant correlations, but observed somewhat lower ADC values in ER highly positive tumors (27). In the majority of recent studies, however, significant correlations between ADC values and estrogen-progesterone receptor positive status were observed (9, 13, 28). This might be explained by the described link between estrogen-progesterone positive status and cellularity of the lesions (22). Additionally, the positive status of estrogen and progesterone receptors alters the perfusion dynamics of the lesions, by inhibition of the angiogenic markers (23). This might also contribute to the observed significant correlations with ADC values, presented in our study (ER- highly positive and PR-negative tumors).

In our study, there were no significant correlations between ADC values and HER2- receptor status (*F* = 0.481, *p* = 0.697). HER2 receptor positive status influences the invasive features of the lesion, cell proliferation, and ability to metastasize. Recent study by Rabasco et al. presented no significant correlations of ADC values with HER2 receptor status, but this lack of correlation was explained by the small study sample (9). In our study sample, we also failed to present any significant correlation with this biomarker. Park et al. showed that ADC values were significantly higher in HER2 positive invasive ductal carcinomas (correlation coefficient = 0.218) in the group of 110 patients, compared to HER2-negative tumors (26). This might come as a surprise, since lower ADC values would be an expected finding in biologically more aggressive, Her2-positive tumors.

Finally, no significant correlations were detected between Ki67 index and ADC values (χ^2^ = 8.22, *p* = 0.222). This observation is concordant with recent research, that revealed that Ki67 index has no impact on the prognosis, and, in addition, no correlations with ADC values were observed (9, 29). Most of the studies that presented no correlations with Ki67 index measured mean ADC values. However, study by Kato et al. presented weak but significant correlation between minimum ADC values and Ki67 index, especially in Luminal B (HER2-negative) tumors. Minimum ADC values of Luminal A were significantly higher than those in Luminal B type, reflecting the differences between Ki67 expressions in those two tumor types (30). In our study, no significant differences between ADC values of different subtypes of tumors were observed, even though the mean ADC values were the lowest in Luminal A type (0.55 × 10^−3^ mm^2^/s), and the highest in the basaloid type (0.88 × 10^−3^ mm^2^/s).

Finally, our study was performed on a 3T scanner. This has certainly contributed to the better spatial resolution and higher signal-to-noise ratio, important for the evaluation of morphologic and kinetic features of the lesions. However, recent comparative studies showed no significant differences in ADC values obtained on 1.5T and 3T scanners, making the choice of the scanner more easy (14).

The current study has some limitations. The major limitation is the retrospective character of the study that was additionally performed in a single institution. The second limitation is a relatively small study sample, which could have contributed to the lack of significance in some correlations. The inclusion of a larger study sample might have resulted in some correlations reaching the level of significance (primarily with ER-expression). Despite the numbered limitations, we believe that our study presented very accurate ADC values in a variety of breast tumor lesions, and adds significantly to the existing body of knowledge.

## Conclusions

In conclusion, according to the results of our study, DWI represents a clinically useful tool for differentiation of malignant from benign lesions given that mean ADC values in benign lesions are significantly higher than those in malignant ones. The cut off ADC values were determined for distinguishing benign and malignant lesions. The cut off value for benign lesion is slightly higher than those reported in the recent literature, due to the fact that we included variety of benign findings, currently observed in the clinical practice, in the analysis. In our opinion, it is important to be aware that high amount of fibrous tissue (observed in fibrosclerosis, fibrous Fas, and inflammation) can reduce the mean ADC values, and thus create a diagnostic bias, if only this parameter was taken into account. Finally, there were significant correlations between mean ADC values and highly ER positive tumors, as well as between mean ADC values and PR negative tumors. This leads to the conclusion that ADC values have some implications in determination of the biological nature of the malignant lesions, that in the future have to be more thoroughly explored.

## Data Availability Statement

The datasets generated for this study are available on request to the corresponding author.

## Ethics Statement

The studies involving human participants were reviewed and approved by Ethical Committee of Faculty of Medicine Novi Sad. The patients/participants provided their written informed consent to participate in this study.

## Author Contributions

JM and JB drafted the manuscript. JM, TI-K, VV-C, DD, and DB-S analyzed the data. JM performed statistical analysis. JM and DB-S revised the manuscript for intellectual content.

### Conflict of Interest

The authors declare that the research was conducted in the absence of any commercial or financial relationships that could be construed as a potential conflict of interest.
